# Therapeutic Drug Monitoring of Everolimus Using Volumetric Absorptive Microsampling and Quantitative Dried Blood Spot Methods with LC-MS/MS in Adult Solid Organ Transplant Recipients: An Analytical and Clinical Comparative Study

**DOI:** 10.3390/molecules30153139

**Published:** 2025-07-26

**Authors:** Arkadiusz Kocur, Bartosz Olkowski, Mateusz Moczulski, Dorota Miszewska-Szyszkowska, Olga Maria Rostkowska, Katarzyna Polak, Katarzyna Korniluk, Teresa Bączkowska, Magdalena Durlik, Tomasz Pawiński

**Affiliations:** 1 Department of Drug Chemistry, Pharmaceutical and Biomedical Analysis, Medical University of Warsaw, Banacha 1, 02-097 Warsaw, Poland; mmoczulski8@gmail.com (M.M.); tomasz.pawinski@wum.edu.pl (T.P.); 2 Students’ Scientific Club by the Department of Transplantation, Immunology, Nephrology and Internal Medicine, Medical University of Warsaw, 02-006 Warsaw, Poland; bartosz.olkowski@icloud.com (B.O.); polak.katarzyna28@gmail.com (K.P.); 3 Department of Transplantation, Immunology, Nephrology and Internal Medicine, Medical University of Warsaw, 02-006 Warsaw, Poland; dorota.szyszkowska@uckwum.pl (D.M.-S.); olga.rostkowska@wum.edu.pl (O.M.R.); teresa.baczkowska@wum.edu.pl (T.B.); magdalena.durlik@wum.edu.pl (M.D.); 4 Metabolism Disorders and Therapeutic Drug Monitoring Laboratory, Medical University of Warsaw Clinical Center, Żwirki i Wigury 63A, 02-091 Warsaw, Poland; katarzyna.korniluk@uckwum.pl

**Keywords:** everolimus, qDBS, VAMS, LC-MS/MS, solid organ transplantation

## Abstract

Everolimus (EVE), an mTOR inhibitor, is widely used in solid organ transplantation (SOT) because of its immunosuppressive properties. Due to its narrow therapeutic window and significant pharmacokinetic variability, therapeutic drug monitoring (TDM) is essential for achieving optimal outcomes. We developed and thoroughly validated a robust LC-MS/MS method to measure EVE levels in venous whole blood (WB) and capillary blood collected using two microsampling devices: Mitra™ (volumetric absorptive microsampling, VAMS) and Capitainer^®^ (quantitative dried blood spot, qDBS). The validation followed EMA and IATDMCT guidelines, assessing linearity (1.27–64.80 ng/mL for WB and 0.50–60 ng/mL for VAMS/qDBS), as well as selectivity, accuracy, precision, matrix effects, recovery, stability, and incurred sample reanalysis. Clinical validation involved 66 matched samples from 33 adult SOT recipients. The method demonstrated high accuracy and precision across all matrices, with no significant carryover or matrix interference. Statistical analysis using Passing–Bablok regression and Bland–Altman plots showed excellent agreement between the microsampling methods and the venous reference. Hematocrit effects were tested both in laboratory conditions and on clinical samples and were found to be negligible. This study provides the first comprehensive analytical and clinical validation of the Mitra and Capitainer devices for EVE monitoring. The validated LC-MS/MS microsampling method supports decentralized, patient-centred TDM, offering a reliable alternative to conventional blood sampling in transplant care.

## 1. Introduction

Everolimus (EVE; brand names: Afinitor^®^, Certican^®^, Votubia^®^, Zortress^®^) is an inhibitor of the mammalian target of rapamycin (mTOR), primarily used as an immunosuppressive agent in therapeutic protocols for solid organ transplantation (SOT). Beyond its immunosuppressive role, EVE is also indicated for use in oncology and in the treatment of tuberous sclerosis complex (TSC) [1].

EVE has a narrow therapeutic index and exhibits significant interindividual variability in both pharmacokinetics and pharmacogenomics. This variability is primarily attributed to its metabolism by the cytochrome P450 enzyme CYP3A4 and its transport by *p*-glycoprotein (*p*-gp) [1,2]. Due to these properties, therapeutic drug monitoring (TDM) is essential for optimizing EVE therapy. Adjusting doses based on blood concentration levels and observed adverse effects is critical for achieving both efficacy and safety. For routine TDM, whole blood—preferably collected in K_2_- or K_3_-EDTA tubes—is the recommended matrix, due to EVE’s high intracellular partitioning into red blood cells [1,2].

The primary objectives of TDM in EVE therapy include identifying sub-therapeutic or supra-therapeutic drug levels, assessing patient adherence, early detection of drug-related toxicity, and individualized dose optimization during treatment initiation. The trough concentration (C_0_) is generally used as a surrogate for systemic exposure, as EVE exhibits linear pharmacokinetics within the therapeutic range [1,2]. The recommended therapeutic trough concentration (C_0_) of everolimus varies depending on the type of SOT. In kidney transplant recipients, the target range is 6–10 ng/mL when used as monotherapy (without tacrolimus or cyclosporine) or 3–8 ng/mL when combined with reduced doses of these calcineurin inhibitors. In liver transplant patients, the therapeutic range is 5–12 ng/mL without tacrolimus or cyclosporine, or ≥3 ng/mL when administered in conjunction with reduced-dose concomitant therapy [1,2].

Recently, patient-centric approaches in the TDM of immunosuppressive agents have emphasized the utility of alternative sampling methods, such as dried blood spots (DBS), quantitative dried blood spots (qDBS), and volumetric absorptive microsampling (VAMS) [1,2,3,4,5]. These techniques offer several advantages, including minimal invasiveness, cost-effectiveness, simplified logistics, and the ability to self-sample outside clinical settings. This decentralization is particularly valuable for transplant recipients living in remote areas with limited access to specialized transplant centres [3].

Among these, VAMS and qDBS technologies stand out because of their reduced susceptibility to hematocrit-related variability, a well-known limitation of traditional DBS methods. Hematocrit levels can affect analyte recovery, spot size, and distribution, potentially leading to inaccuracies in quantification. Volumetric microsampling largely mitigates this issue by delivering fixed blood volumes; however, the impact of hematocrit should still be evaluated for each analyte individually during method development and validation [1,2,3,4,5]. Currently, several devices based on volumetric microsampling technologies are available in the diagnostic market, including [1,3,4,5,6,7]:

A. Volumetric absorptive microsampling (VAMS) devices:

- *Mitra™* (Neoteryx, Torrance, CA, USA)
- *Tasso™* (Tasso Inc., Seattle, WA, USA)

B. Quantitative dried blood spot (DBS) devices:

- *Capitainer^®^* (Capitainer AB, Solna, Sweden)
- *hemaPen™* (Trajan Scientific and Medical, Ringwood, Australia)
- *HemaXis^®^* (DBS System SA, Gland, Switzerland)

**Figure 1:**
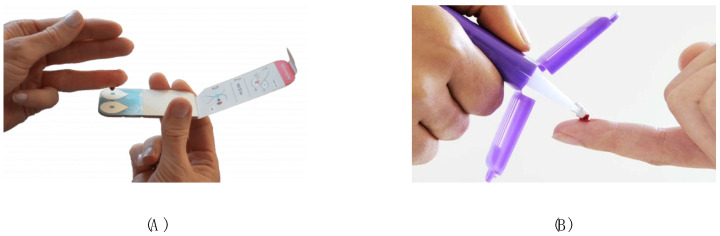
Capitainer^®^ ((**A**); quantitative dried blood spot) and Mitra™ ((**B**); volumetric absorptive microsampling) devices (photos presented with permission from the manufacturers) [6,7]. The Capitainer B^®^ is equipped with two inlets and two Ahlstrom 222 paper spots to generate two independent samples. The Mitra™ device is a polymer tip embedded in a plastic handler. Both systems were calibrated to collect 10 µL of capillary blood (with RSD < 5%).

The two most popular approaches based on microsampling strategies, Mitra™ and Capitainer^®^, have been compared in the presented study (device visualization presented in Figure 1). 

The specific strengths and limitations of the tested devices are analyzed in Table 1. Additional information about the strengths, limitations, device-specific characteristics, and practical considerations associated with these technologies is described in detail in recent publications [3,4].

The primary aim of this study was to develop and fully validate a novel LC-MS/MS method for quantifying everolimus (EVE) in venous whole blood and capillary blood samples, using two independent volumetric microsampling devices: Mitra™ and Capitainer^®^. Each device enables the collection of 10 µL of capillary blood. The secondary objective was to clinically evaluate this method in adult solid-organ transplant recipients receiving EVE therapy. Analytical performance was assessed through cross-validation and clinical validation, with particular attention to the potential impact of the haematocrit effect. To the best of our knowledge, this is the first report to present both analytical and clinical comparisons of the Mitra™ and Capitainer^®^ devices for routine therapeutic drug monitoring of everolimus.

## 2. Results

### 2.1. Method Development and Optimization

In this study, an LC-MS/MS method for the quantification of EVE in venous whole blood (WB) and capillary blood (CB) collected using two independent microsampling devices—Mitra™ (VAMS) and Capitainer^®^ (qDBS)—was optimized and validated.

D_4_-everolimus (D_4_-EVE) was employed as the internal standard (IS) in accordance with EMA guidelines, which recommend the use of stable isotope-labelled ISs when available [8]. Although ^13^C,D_2_-EVE is commercially accessible, our evaluations indicated greater interference of ^13^C,D_2_-EVE due to cross-interferences with non-labelled everolimus (16.37 ± 2.58%, n = 3), making D_4_-EVE the preferred choice, for which the observed interferences were lower (1.76 ± 0.21%, n = 3). The mentioned interferences were expressed as the EVE peak area to SIL-IS peak area per cent ratio in a blank sample spiked with IS to obtain a final concentration of 10 ng/mL.

Whole blood samples were prepared using a protein precipitation protocol involving a mixture of zinc sulfate, methanol, and acetonitrile, which is a widely accepted method for immunosuppressant extraction in LC-MS/MS assays [2,3,4]. The addition of methanol improved the miscibility between the aqueous and organic phases. Following sample vortexing, a cold precipitation solution was added, and the mixture was frozen prior to centrifugation. This process enhanced analyte recovery, reduced matrix effects, and produced a clear supernatant suitable for LC-MS/MS analysis.

Mitra sample preparation was based on our previously published protocols, with extraction performed using pure methanol [3,4]. As with WB samples, incubation at low temperatures significantly improved the extract clarity and LC-MS/MS signal quality.

In contrast, Capitainer^®^ samples require a more rigorous extraction procedure because of matrix-related interferences previously reported for this device [9,10,11,12]. To enhance purification, an additional step incorporating MgSO_4_ and sodium acetate (CH_3_COONa) was introduced. Acetonitrile was used to extract the dried matrix from quantitative dried blood spots (qDBS), facilitating effective phase separation and cleaner extracts for analysis.

**Figure 2:**
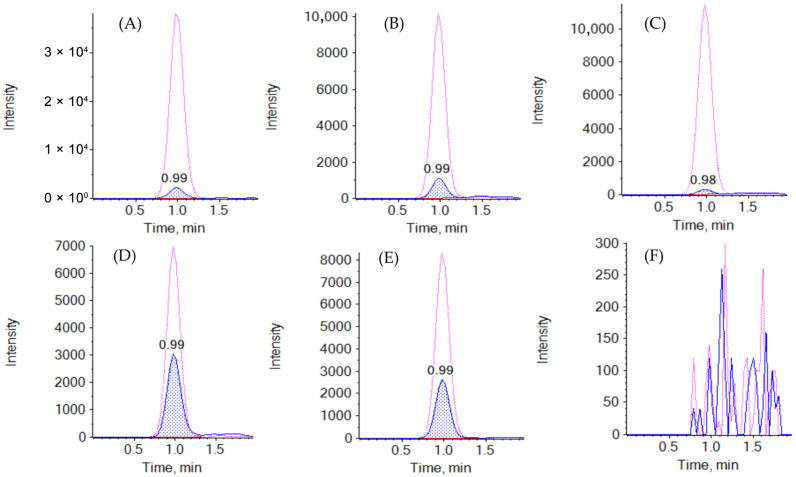
Representative chromatograms of EVE (blue peak, retention time = 1.05 min) and corresponding SIL-IS (EVE-D_4_, pink line, retention time = 1.05 min). (**A**–**C**) represent the LLOQ in whole blood, VAMS, and qDBS samples, respectively. (**D**,**E**) represent clinical samples collected from patients (determined EVE concentrations: 3.985 and 4.321 ng/mL in VAMS and qDBS, respectively). A double blank chromatogram for a whole blood sample is presented on panel (**F**).

For chromatography, different analytical columns were tested, but ultimately, a polar-modified C_18_ column was used (Kinetex Polar C_18_), which allowed for the reduction in the EVE retention time. The buffering of mobile phases with formic acid and ammonium acetate (0.1% and 4 mM of final content) in combination with gradient mode ensured appropriate peak shape and analyzed compound retention time (~0.92 min). Representative chromatograms are shown in Figure 2. The mass spectrometry parameters were optimized using direct syringe infusion of EVE and D_4_-EVE solutions (diluted with B mobile phase to each concentration of 5 ng/mL). 

### 2.2. Validation Results: Determination of EVE in Whole Blood

The LC-MS/MS method developed for EVE quantification in whole blood was validated over a calibration range of 1.27–64.80 ng/mL. Linearity was assessed using 10 independent calibration curves, each consisting of seven concentration levels, with a blank sample (processed with an internal standard, without analyte) and a double blank sample (processed without IS and analyte). The mean calibration equation was set as y = 0.6227x − 0.02702, with a correlation coefficient (R^2^) of 0.9996, using a 1/x calibration-weighting model.

The nominal concentrations of the calibrators and QC samples are provided in Section 4.3. The method exhibited acceptable selectivity following the EMA/FDA criteria [8,13]. No significant interference was observed from endogenous compounds, and any interfering chromatographic peaks remained below 15% of the mean LLOQ peak area (LLOQ = 1.27 ng/mL).

The limit of detection (LOD) was established at 0.13 ng/mL using the lowest calibration standard (CS_1_) dilution with blank whole blood, resulting in a signal-to-noise ratio (S/N) greater than 50. Accuracy and precision were evaluated at five QC levels (LLOQ, LQC, MQC_1_, MQC_2_, and HQC) in six replicates under both intra-day and inter-day conditions. All values met the EMA acceptance criteria, with CV% values below 10%, in concordance with the IATDMCT guidelines (Table 2) [2,8,13,14].

The potential carry-over effect was assessed by injecting a blank sample immediately following a high QC sample in a single analytical run. No significant carry-over was identified, with mean with SD responses of 0.97 ± 1.08% for EVE and 0.33 ± 0.61% for EVE-D_4_ (n = 10). All measurements met these acceptance criteria, confirming the absence of significant carry-over effects in the validated method [8,13]. The matrix effect (ME), absolute recovery (AR), and process efficiency (PE) were evaluated according to the protocols of Taylor et al. and Matuszewski et al. [15,16]. The matrix effect, normalized to the IS, was below 15% for both LQC and HQC levels (n = 6; Table 3), indicating minimal ion suppression and an appropriate extraction recovery rate. 

Based on the well-described stability of EVE in whole blood, stability testing was focused on the bench-top and autosampler stability of the extracts (samples after preparation). EVE remained stable throughout the extraction procedure (bench-top) and after 7 days of storage at 4 °C in the autosampler (Table 4). The analyte has been considered stable when the determined concentration at any tested time point was within ±15% of the value determined initially (T = 0). Additionally, whole blood calibrators and QC samples appeared to be stable after three freeze–thaw cycles (data provided by manufacturers: Chromsystems and RECIPE in appropriate manuals) [17,18].

### 2.3. Validation Results: Determination of EVE in Mitra™ (VAMS) and Capitainer^®^ (qDBS)

The LC-MS/MS methods of EVE quantification in VAMS and qDBS samples were validated across a 0.50–60 ng/mL calibration range using seven calibration levels. The nominal concentrations of the calibrators and quality control (QC) samples are provided in Section 4.3. The mean calibration equation was established based on 10 calibration curves: y = 0.08093x + 0.00374 (R^2^ = 0.9989) for VAMS, and y = 0.07539x + 0.00935 (R2 = 0.9959). Both validated microsampling-based methods demonstrated no interference from endogenous analytes or co-administered compounds. All chromatographic responses attributed to potential interferences were below 15% of the mean LLOQ peak area (0.50 ng/mL) [8,13]. The limit of detection (LOD) was established at 0.15 ng/mL, based on the experimental analysis of the S/N ratio greater than 50 thresholds. Accuracy and precision were evaluated for Mitra™ and Capitainer^®^ samples at five QC levels (LLOQ, LQC, MQC_1_, MQC_2_, and HQC), as defined in Section 4.3, across intra- and inter-day experiments (n = 6 per level). The calculated values fully met the EMA acceptance criteria, with the coefficient of variation (CV%) consistently below 10%, in agreement with the IATDMCT guidelines (Table 2) [2,8,14]. No significant carry-over was observed, with mean background signals of 0.25 ± 0.43% for EVE and 0.33 ± 0.61% for EVE-D_4_ in the case of VAMS sample validation. For the Capitainer^®^ samples, the acceptance criteria were also fulfilled: mean background signals of 0.31 ± 0.50% for EVE and 0.29 ± 0.28% for EVE-D_4_. The analyte in both devices was stable during 6 months of storage at ambient room temperature in zip bags with desiccant. Similarly to the whole blood pretreatment protocol, EVE remained stable throughout sample preparation and during post-extraction storage at 4 °C for 7 days for both tested devices (Table 3). Additional tests were performed to evaluate EVE stability in the dried matrix under various conditions, as shown in Table 3. Both VAMS and qDBS devices remained stable for 6 months at room temperature and 40 °C (storage in sun-protected zip bags with desiccant). After storage at 60 °C, the analyte remained stable in the VAMS and qDBS devices for 24 h at LQC levels and 36 h at HQC levels (n = 3). Due to the reduced stability of the analyte at 60 °C, the test was stopped after 36 h, as the EVE was unstable in LQC samples at that time point. Consequently, lower stability was expected at later time points. The freeze–thaw experiment was not conducted for microsamples (calibrators and quality controls) due to their specific storage conditions (room temperature in zip bags with desiccant). The matrix effect (ME), absolute recovery (AR), and process efficiency (PE) were evaluated according to the protocols published by Matuszewski et al. and Taylor et al. [15,16]. The matrix effect, normalized to the internal standard, remained within ±15% at both the LQC and HQC levels (n = 6), confirming minimal ion suppression and efficient extraction of analyte and compensation of ME for both tested devices (Table 4).

### 2.4. Clinical Results and Demographic Data

A total of 33 adult patients who had undergone solid organ transplantation at the Transplant Medicine and Nephrology Outpatient Clinic, Infant Jesus Teaching Hospital, Medical University of Warsaw Clinical Centre, were enrolled in the study. Among them, 12.12% had received at least two transplants. In total, 66 triplicate sample sets were collected simultaneously from each participant for everolimus (EVE) analysis, comprising venous whole blood (WB), capillary volumetric absorptive microsampling (VAMS), and quantitative dried blood spot (qDBS).

The study was to be conducted between March 2024 and June 2025. The basic demographic and clinical characteristics of the study cohort are summarized in Table 5. In most cases, immunosuppressive regimens initially included tacrolimus or cyclosporin in combination with mycophenolate mofetil and corticosteroids. Everolimus was introduced to facilitate calcineurin inhibitor (CNI) dose reduction, primarily in response to BK virus-associated nephropathy (BKVN) or post-transplant malignancy.

### 2.5. Clinical Application of Validated Methods

Clinical samples collected in triplicate from each enrolled patient (see Section 2.4) were analyzed using validated LC-MS/MS methods for EVE quantification. The mean EVE concentrations obtained for each sample type were as follows:

- Venous whole blood (WB): 4.696 ± 1.967 ng/mL (range: 0.800–10.895 ng/mL),
- Volumetric absorptive microsampling (VAMS): 4.707 ± 1.960 ng/mL (range: 0.710–10.895 ng/mL),
- Quantitative dried blood spots (qDBS): 4.820 ± 2.118 ng/mL (range: 0.877–11.897 ng/mL).

**Figure 3:**
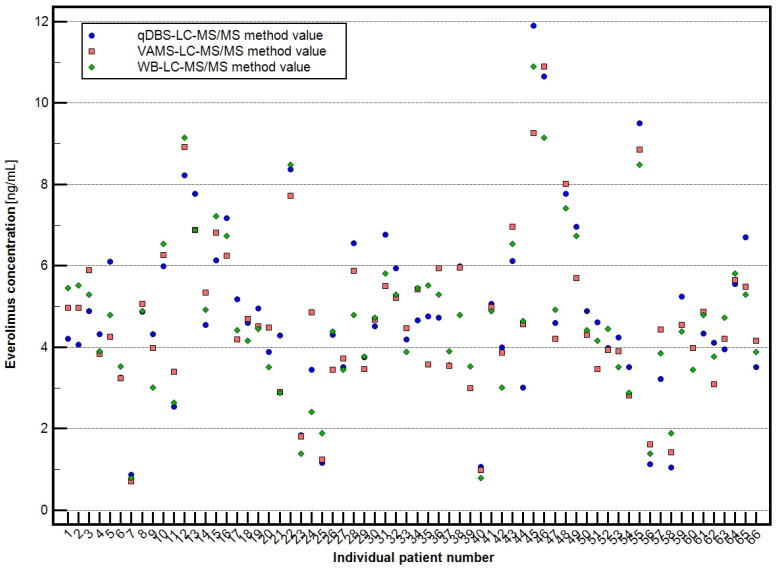
Summarized results of everolimus LC-MS/MS quantification in whole blood (WB), volumetric absorptive microsampling (VAMS), and quantitative dried blood spot (qDBS) samples.

The summarized variables are presented as a multi-point graph in Figure 3. 

### 2.6. Cross-Validation and Clinical Evaluation

**Figure 4:**
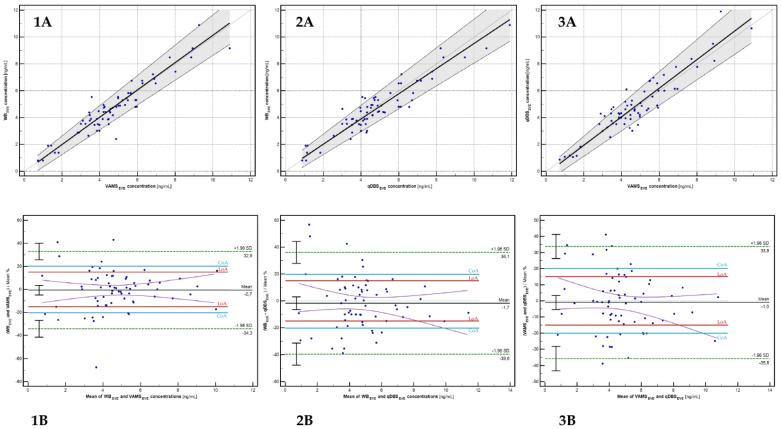
Graphical results of method comparison using Passing-Bablok regression model ((**1A**–**3A**) panels) and Bland–Altman parametric bias estimation ((**1B**–**3B**) panels). Compared relationships: (**1A**,**1B**)—WB_EVE_ versus VAMS_EVE_; (**2A**,**2B**)—WB_EVE_ versus qDBS_EVE_; (**3A**,**3B**)—VAMS_EVE_ versus qDBS_EVE_. On the Bland–Altman plots, the green line expresses the ±1.96 SD range, the purple line expresses regression, while the blue and red lines mark the clinical limit of agreement (CoA) and analytical limit of agreement (LoA), respectively. The mean value of the estimated Bland–Altman bias is provided as a percentage of the mean value. Explanation: (**1A**).

To assess the clinical equivalence between different blood collection devices for the quantitative determination of EVE, regression and correlation analyses were performed using the Passing–Bablok test and Bland–Altman bias estimation [5,19,20,21]. These statistical methods were used to evaluate the level of agreement and potential bias between EVE concentrations determined using microsampling techniques and the reference venous whole blood method. The results of the method interchangeability, along with additional statistical outputs, are presented in Figure 4 and Table 6.

### 2.7. Hematocrit Effect Evaluation

The hematocrit effect (HE) was experimentally evaluated for both Mitra™ and Capitainer^®^ devices at low (23%), medium (37%), and high (58%) hematocrit levels. Quality control samples at LQC and HQC concentrations were prepared by adding/removing the plasma amount to/from whole blood to achieve the target hematocrit values, followed by spiking with the analyte solution (see Section 2.3). Recovery assessments (n = 3 per level) showed no significant hematocrit-dependent bias, with CV% values for analyte recovery remaining below 15% at all hematocrit levels (appropriate table presented in Appendix A).

**Figure 5:**
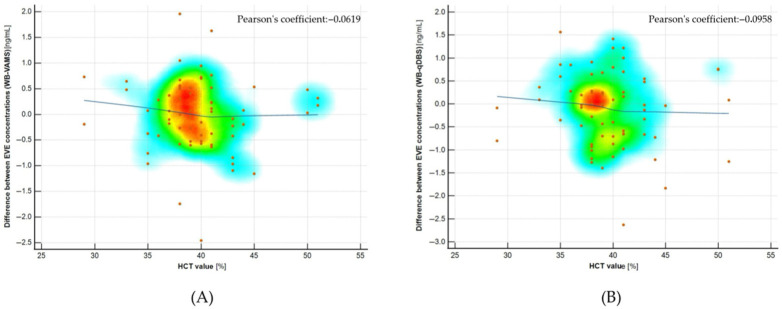
Scatter diagrams presented no significant correlation between individual hematocrit level and difference between everolimus (EVE) concentration determined in reference method and microsampling method: (**A**) EVE whole blood (reference) level–VAMS EVE level, (**B**) EVE whole blood (reference) level–qDBS EVE level. Pearson coefficients, which characterize the relationship, are shown on each graph. Line drift represented the trend of correlation between paired data.

Additionally, the potential influence of hematocrit was evaluated in clinical samples by comparing the measured everolimus concentrations in venous whole blood and microsamples with individual hematocrit (HCT) values, determined using a validated colorimetric assay (Section 4.10). No statistically significant correlations were found between hematocrit levels and the differences in everolimus concentrations across the tested sampling methods, indicating that both microsampling devices provided hematocrit-independent results. A summary of the hematocrit effect evaluation is shown in Figure 5.

## 3. Discussion

The presented study is a continuation of the evaluation of microsampling methodologies in the Polish population undergoing immunosuppressive therapy after solid organ transplantation [3,4]. Although EVE is not routinely used as a first-line agent in post-transplant immunosuppressive regimens, its inclusion remains relevant due to potential scenarios involving drug switching. Consequently, the development and validation of microsampling-based monitoring approaches for EVE are clinically essential and contribute to the expansion of patient-centric therapeutic drug monitoring strategies.

Numerous single- and multi-analyte LC-MS/MS methods for EVE quantification have been published in the literature [1,22]. In the presented study, the validation of the WB-LC-MS/MS method for EVE determination was not the primary objective, but instead served as a reference point for the optimization of microsampling-based methodologies. The protocols described previously mainly involved protein precipitation of organic/non-organic mixtures, with the addition of ascomycin (structurally unrelated to the EVE internal standard) or SIL-IS (EVE-D_4_ or ^13^C,D_4_-EVE). Here, we used the EVE-D_4_, which resulted in a lower signal of EVE compared to ^13^C,D_4_-EVE (1.33% versus 5.42% for a 10 ng/mL IS concentration). That type of interference had been previously reported, as noted by Deprez et al.—decreasing the final SIL-IS concentration in the sample resolved this issue [11]. What is crucial is that our homemade LC-MS/MS method is regularly evaluated using external proficiency testing schemes (LGC, Teddington, UK). 

In the case of microsampling, a few studies have successfully reported the application of non-volumetric dried blood spots (DBS). The main weakness of the DBS application is the necessity of hematocrit estimation in a sample due to the potential hematocrit effect [3,6]. Therefore, volumetric-based techniques are more attractive due to lower amounts of collected matrix, and the sampling process is more straightforward for patients (compared to DBS). Extraction of the analyte from Mitra™ and Capitainer^®^ devices is similar to that in DBS-based applications. For example, in the presented study, MeOH was chosen as the most efficient solvent for EVE extraction from Mitra samples, similarly to the study by Verheijen et al. [23].

In contrast, for qDBS samples, the extraction process of the analyte is more vigorous and complex due to interferences caused by substances in the glue used to spot the device (Patent Blue dye and polyvinyl alcohol). Mentioned interferences were previously reported by Deprez et al. and Vethe et al. [9,10,11]. To avoid ion suppression caused by concomitantly extracted compounds, liquid–liquid extraction (LLE) or salt-assisted liquid–liquid extraction (SALLE) may be used, as recently published studies have shown. In the presented research, we introduced, for the first time, the addition of QuEChERS salts to increase recovery and purification rates. On the other hand, for the extraction of EVE from qDBS, we used acetonitrile to achieve effective separation of phases. Additionally, we have observed that sonication at a temperature of 40 °C for 15 min improved the total recovery of analyte—similar findings were also previously reported by Le et al. [24].

Considering the LC-MS/MS conditions optimized in the presented study, the application of a polar-modified C18 column provides an opportunity for the simultaneous determination of creatinine with EVE. Moreover, the possibility of simultaneously determining other immunosuppressive drugs with EVE was also positively evaluated in our laboratory. The application of the mentioned chromatographic column ensured an attractive retention time (~0.92 min) with a relatively short total run time (2.00 min). A simple gradient mode is highly beneficial in diagnostic laboratories with TDM service.

According to the calibration range, for the WB-LC-MS/MS method, we have used commercially available calibrators and quality control (QC) as an element to simplify the daily practices of the TDM laboratory. The commercially available reference materials (used as whole blood hemolysates) are applicable with the Mitra™ (VAMS) technique, with promising results. On the other hand, the Capitainer^®^ devices require the application of fresh whole blood calibrators/QC spiked with the analyte for the generation of a qDBS spot sample with the appropriate quality. The density of hemolysate differs from that of whole blood, which causes issues with the generation of drops characterized by a higher area. Interestingly, one of the laboratories participated in a pilot proficiency study of microsampling for the determination of immunosuppressive agents, declaring the use of purchased QC for daily practice with the Capitainer^®^ device [25]. It should be highlighted that the manufacturer of the mentioned device recommended the use of fresh blood for validation and method calibration [6]. 

The cross-validation (comparison of paired results with each other) and clinical validation (i.e., evaluation of bias within the clinical limit of agreement) are crucial to ensure and provide interchangeability between compared methods. In the presented study, the Passing–Bablok regression analysis and Bland–Altman bias estimation tests were performed to assess the agreement between capillary microsampling devices (VAMS and qDBS) and the reference venous whole blood (WB) method for everolimus quantification. The regression models demonstrated high concordance between analyzed sample types, with slopes and intercepts indicating minimal proportional and constant bias. Specifically, for VAMS versus WB, the regression equation was EVE_WB_ = 1.017(EVE_VAMS_) − 0.040, with the 95% confidence intervals for both the slope and intercept including the theoretical values of 1 and 0, respectively (Table 5). For qDBS versus WB, the regression function yielded EVE_WB_ = 0.938(EVE_qDBS_) + 0.143, confirming a strong relationship between paired data. These results confirm the equivalence of the microsampling methods with conventional venous sampling, supporting their suitability for the clinical therapeutic drug monitoring (TDM) of everolimus. Similarly, the regression analysis met the acceptance criteria for the qDBS versus VAMS relationship, as determined by the EVE value. In the case of percentage bias estimation, the mean bias was lower than 15%, which confirms that the given acceptance criteria have been met for all analyzed relationships. The analytical (mean bias < 20%) and clinical (mean bias < 15%) thresholds were fulfilled for WB versus VAMS and WB versus qDBS paired results. Interestingly, the acceptable analytical bias (LoA) was confirmed for the qDBS versus VAMS relationship, but not for the clinical acceptance level (CoA). Potentially, the various mechanisms (VAMS versus qDBS) of sample collection between microsampling devices caused differences in results. The Pearson’s coefficient was higher than 0.92 for each tested pair, confirming strong relationships (correlation) between results. In contrast to Verheijen et al., we demonstrated that no correction formula is necessary to recalculate CB EVE levels to estimate EVE concentrations in whole blood for the tested microsampling devices [23]. Our findings, according to cross- and clinical validation of the qDBS device, confirmed the results of the Deprez et al. study [11]. The mean bias estimated using the Bland–Altman test in the mentioned study was 0.5%, whereas in our study, it was 1.73%. It should be noted that cross- and clinical validation in the Deprez et al. study was performed using qDBS samples generated from whole blood samples collected by venipuncture [11]. The Yoo et al. study evaluated the 32 paired EVE concentrations determined in WB and VAMS samples [26]. However, the number of samples included in the final clinical validation was lower than recommended (40 paired results), and the strong relationship evaluated in Deming regression has been demonstrated, with a mean bias of −0.79 ng/mL estimated using Bland–Altman analysis [26]. In Paniagua-Gonzales’ study, the EVE was included in the validation of the LC-MS/MS method; however, no VAMS samples from patients undergoing EVE treatment were collected [27]. In the Gruzdys et al. study, the clinical feasibility of microsampling for EVE determination was proven using the satisfactory results of Deming regression only [28]. Based on the above, it appears that our study is the first to provide complete cross- and clinical validation of EVE in VAMS and qDBS capillary blood microsamples. 

The absent hematocrit effect influence in the presented study has been evaluated using two approaches: first, using spiked whole blood samples with various HT levels, and second, analyzing differences in EVE levels between VAMS and WB samples correlated with individual HT values. The absence of a hematocrit influence was also demonstrated by Deprez et al. [11]. The total recovery of the analyte from qDBS spots (Capitainer B^®^ device) ranged from 85% to 115% across a wide range of hematocrit levels (0.18–0.61) [11]. In the Yoo study, the hematocrit influence was considered negligible due to similar hematocrit levels in paired whole blood and VAMS (Mitra™ device) samples (across the 29.8–49.3% range) [26]. Similar findings were reported in Paniagua-Gonzales et al.’s work, where differences in analyte recovery (normalized to the internal standard) across hematocrit values of 0.20–0.62 were in the 85–115% range [27].

The presented study has some limitations. First, the study population was enrolled from a single transplant centre only. Additionally, no self-sampling in a patient’s scenario has been tested, especially without the supervision of qualified medical personnel. However, the brief interview and preliminary explanation of the sampling process using both devices, along with the successful enrollment of patients after using both devices, confirm the promising feasibility of this approach in adult SOT recipients. Although our study focused on the analytical and clinical comparison of VAMS and qDBS-based devices, further studies should investigate potential errors associated with the home-based self-sampling process. Valuable studies recently published by Boffel et al. and Vethe et al. have confirmed the importance of feasibility evaluation using microsampling devices, especially in vulnerable populations, such as adolescents [10,29]. Therefore, before applying our method to home-based self-sampling, the extensive training of patients and evaluation of sampling without supervision should be carefully performed. The stability studies confirmed satisfactory stability under standard conditions, meeting the logistics process requirements for the shipment and storage of samples. 

## 4. Materials and Methods

### 4.1. Reagents, Chemicals, and Laboratory Equipment

The reference standard of everolimus (EVE; C_53_H_83_NO_14_; ≥99.85% purity, catalog no. HY-10218) was purchased from MedChemExpress LLC (Sollentuna, Sweden). The stable isotope-labelled internal standard (SIL-IS), D_4_-everolimus (≥99% of deuterium incorporation), was obtained from Cayman Chemical (Ann Arbor, MI, USA).

LC–MS grade solvents, including water, acetonitrile, methanol, and 2-propanol, as well as mobile phase additives (ammonium acetate and formic acid), were acquired from Merck (Darmstadt, Germany). Zinc sulfate solution (0.5 M, titration grade) was sourced from Chempur (Piekary Śląskie, Poland). CHROMABOND^®^ pre-weighted QuEChERS salt mix II (6000 mg MgSO_4_, 1500 mg CH_3_COONa) was supplied by Macherey-Nagel (Dueren, Germany). A colorimetric assay kit for hemoglobin determination was also obtained from Cayman Chemical (Ann Arbor, MI, USA) [30].

Drug-free whole blood for method development and validation was collected from healthy volunteers in the Regional Centre of Blood Donation and Hemotherapy (Warsaw, Poland). Volumetric absorptive microsampling (VAMS) devices—10 µL Mitra™ tips—were obtained from Neoteryx (Torrance, CA, USA) via Bioanalytic (Gdańsk, Poland). Quantitative dried blood spot (qDBS) cards (Capitainer B^®^), enabling 10 µL blood collection, were provided by Capitainer AB (Solna, Sweden).

Basic laboratory equipment, including pipettes, tips, and conical and reaction test tubes, was provided by Eppendorf (Hamburg, Germany). Polypropylene vials with reduced-volume corning inserts were sourced from J.G. Finneran (Vineland, NJ, USA).

### 4.2. Stock, Working Solutions of Everolimus, and Internal Standard

Two independent primary stock solutions of everolimus (EVE) were prepared gravimetrically at a concentration of 1 mg/mL using a methanol/water mixture (50:50, *v*/*v*) as the solvent. The preparation of two separate stock solutions using different initial weights was performed in accordance with EMA guidelines, which recommend the independent preparation of calibration and quality control solutions [8].

Intermediate dilutions were subsequently prepared at concentrations of 100 mg/L and 10 mg/L, and further diluted with a methanol/water (50:50, *v*/*v*) mixture to generate working solutions at 1000 ng/mL and 100 ng/mL, respectively.

The solid internal standard (SIL-IS), D_4_-everolimus (EVE-D_4_), was dissolved in methanol to produce a stock solution at a concentration of 5 mg/L. This was further diluted to prepare an intermediate solution of 0.5 mg/L, followed by working solutions of 0.05 mg/L for whole blood and 0.01 mg/L for microsample analysis.

All prepared solutions were stored in amber conical tubes at –40 °C and remained stable for up to one year. 

### 4.3. Calibrators (CS) and Quality Control (QC) Preparation

For the validation of the LC-MS/MS method for EVE quantification in whole blood, a commercial seven-point calibrator set (CS) was obtained from RECIPE Chemicals + Instruments GmbH (Munich, Germany). The assigned EVE concentrations for each calibrator level are presented in Table 7. To enhance method reliability, four-level commercial quality control (QC) materials (levels I–IV) were sourced from Chromsystems Instruments & Chemicals GmbH (Gräfelfing, Germany), with nominal concentrations provided in Table 8. 

In contrast, for microsampling-based method validation, both CS and QCs were prepared in-house. Fresh whole blood (45 µL) was spiked with 5 µL of the corresponding working solution to achieve the target concentrations defined in Table 7 and Table 8. The samples were gently vortexed for 15 s and then incubated at 37 °C for 1 h to allow the analyte to equilibrate. After a second vortexing step, 45 µL of the spiked sample was used to load Mitra™ tips (Neoteryx, Torrance, CA, USA) at a 45° angle, ensuring complete saturation of the absorptive matrix (entirely red colour of the tip). For Capitainer B^®^ devices (Capitainer AB, Sweden), 25 µL of spiked whole blood was transferred to the sample inlet to ensure spot generation characterized by appropriate volume. Through an internal microfluidic system in the device, 10 µL of blood was deposited onto the Ahlstrom 222 paper collection zone. Both Mitra™ tips and Capitainer B^®^ cards were dried in light-protected aluminum zip-lock bags containing desiccant for a minimum of 1 h. Once dried, the devices were removed using tweezers and processed according to the analytical protocol described in Section 4.5. It is important to note that Capitainer AB recommends using fresh whole blood, rather than hemolysate-based materials, for the preparation of calibrators and controls [6]. Therefore, in-house-generated CS and QC samples were used exclusively for the validation of microsampling-based methods.

### 4.4. Clinical Samples Collection

The clinical component of this study was carried out at the Transplant Medicine and Nephrology Outpatient Clinic of the Infant Jesus Teaching Hospital, which is part of the Medical University of Warsaw Clinical Centre. This facility belongs to the Department of Transplantology, Immunology, Nephrology, and Internal Diseases at the Medical University of Warsaw, Poland. Whole blood (WB) and capillary blood samples (collected using VAMS and qDBS) were obtained simultaneously from adult SOT recipients before they received their daily dose of Certican^®^ (Novartis, Basel, Switzerland). A trained nurse drew venous blood (2.4 mL) via standard phlebotomy into K_3_-EDTA tubes (Sarstedt, Nümbrecht, Germany). At the same time, capillary blood was collected by fingerstick using a hematology lancet (Sarstedt, Nümbrecht, Germany), followed by the generation of paired microsamples with Mitra™ (2 × 10 µL) and Capitainer B^®^ (2 × 10 µL) devices. All microsampling tools were stored in aluminum zip-lock bags with desiccant at room temperature prior to analysis. Manufacturer-recommended procedures were adhered to during sample collection and storage. The demographic and clinical information of the study population is detailed in Section 2.5. The study received approval from the Bioethical Committee of the Medical University of Warsaw (Approval No. 4/2024, dated 8 January 2024). Written informed consent was obtained from all participants following a comprehensive explanation of the study protocol. The research adhered to the Declaration of Helsinki and Good Clinical Practice (GCP) standards.

### 4.5. Samples Preparation Protocol

#### 4.5.1. Whole Blood Samples

A 50 µL aliquot of calibrator (CS), quality control (QC), or patient whole blood sample was reversely pipetted into a 1.5 mL Eppendorf-type transparent microcentrifuge tube. Subsequently, 10 µL of internal standard solution (EVE-D_4_, final concentration: 10 ng/mL in whole blood sample) was added, and the sample was vortexed for 15 s (Ohaus, Parsippany, NJ, USA). Next, 300 µL of a cold protein precipitation mixture (0.5 M zinc sulfate: water: acetonitrile: methanol, 10:40:25:25, *v*/*v*/*v*/*v*) was added and vortexed for 15 min at 800 RPM (Endeavor™ 5000 vortex, Ohaus). The sample was then incubated at –40 °C for 15 min, followed by centrifugation at 16,873× *g* for 5 min. A 100 µL aliquot of the clear supernatant was transferred to a polypropylene vial with reduced-volume inserts, and 1 µL was injected into the LC-MS/MS system.

#### 4.5.2. Mitra™ (VAMS) Samples

A dried Mitra™ tip was removed from the plastic holder using tweezers and placed into a 2 mL Eppendorf-type tube. Then, 150 µL of pure methanol and 10 µL of internal standard solution (EVE-D_4_, final concentration: 10 ng/mL in dried blood) were added. The tube was placed in an ultrasonic water bath at 40 °C for 15 min (40 kHz; Power Sonic 510, Hwashin Technology, Seoul, Republic of Korea). Subsequently, 150 μL of the cold protein precipitation mixture (same as above) was added, and the sample was vortexed for 15 min at 800 RPM. After incubation at –40 °C for 15 min, the sample was centrifuged at 16,873× *g* for 10 min. A 100 µL portion of the supernatant was transferred to a polypropylene vial with volume-reduced inserts, and 5 µL was injected into the LC-MS/MS system.

#### 4.5.3. Capitainer^®^ (qDBS) Samples

The dried blood spot was carefully removed from the Capitainer B^®^ card using device-specific tweezers (Capitainer AB, Solna, Sweden) and transferred into a 2 mL Eppendorf-type tube. The extraction procedure was identical to that used for Mitra™, except that acetonitrile was used in place of methanol during the initial matrix extraction step. Following incubation at –40 °C for 15 min, ~30 mg of QuEChERS salt mixture (MgSO_4_: CH_3_COONa; 6:1.5; *w*/*w*) was added. The sample was vortexed for 1 min and then centrifuged at 16,873× *g* for 10 min. The upper organic phase (100 µL) was carefully transferred to a chromatographic polypropylene vial, and 5 µL was injected into the LC-MS/MS system.

### 4.6. Chromatographic and Mass Spectrometry (LC-MS/MS) Conditions

For this study, quantitative analysis of EVE was performed using a Triple Quad™ 6500 mass spectrometer (Applied Biosystems, Framingham, MA, USA) coupled to an Exion LC™ system. The mass spectrometer was operated in positive electrospray ionization (ESI+) mode using multiple reaction monitoring (MRM) to ensure the appropriate selectivity of target analytes detection (see Table 9 for transitions).

Ion source parameters were set as follows: source temperature, 300 °C; ion spray voltage, +5.50 kV; curtain gas, 40 arbitrary units; source gas 1 (GS_1_), 60 arbitrary units; and source gas 2 (GS_2_), 30 arbitrary units. Instrument optimization and MRM transitions were defined to ensure high sensitivity and selectivity for both everolimus and corresponding internal standard (D_4_-EVE).

Chromatographic separation was performed using a Kinetex^®^ Polar C_18_ column (50 × 2.1 mm, 2.6 µm) linked with a SecurityGuard Ultra™ pre-column (Phenomenex, Torrance, CA, USA). The column oven temperature was maintained at 60 °C, and the flow rate was set at 0.7 mL/min. The total run time was established as 2.0 min, with a gradient elution programme as follows:

- 0.01–0.50 min: 50% mobile phase A/50% mobile phase B;
- 0.51–1.49 min: 5% A/95% B;
- 1.50–2.00 min: 50% A/50% B (re-equilibration).

Mobile phase A consisted of LC–MS-grade water containing 4 mM ammonium acetate and 0.1% formic acid. Mobile phase B was a 50:50 (*v*/*v*) mixture of acetonitrile and methanol, modified with 4 mM ammonium acetate and 0.1% formic acid. The compressibility of mobile phases A and B was set to 0.45 and 1.25 GPa^−1^ respectively.

Samples were stored at 4 °C in the autosampler before injection. The injection volume was set to 1 μL for whole blood samples and 5 μL for microsample-based methods. The LC needle was rinsed between runs using an internal/external wash protocol (a mixture of water: MeOH: ACN: IPA with 0.1% formic acid) to prevent carry-over.

### 4.7. Analytical Validation Methodology

The development and validation of the multi-matrix LC-MS/MS method for EVE quantification were carried out at the Department of Drug Chemistry, Pharmaceutical and Biomedical Analysis, Medical University of Warsaw (Warszawa, Poland). The validation process adhered to current regulatory guidelines issued by the European Medicines Agency (EMA) and the U.S. Food and Drug Administration (FDA) [8,13]. Given the use of microsampling techniques and the immunosuppressive nature of the analyte, relevant recommendations from the International Association of Therapeutic Drug Monitoring and Clinical Toxicology (IATDMCT) were also considered [2,14].

The method was validated for the quantification of EVE in both venous whole blood and capillary microsamples. Validation parameters included assessment of selectivity, specificity, lower limit of quantification (LLOQ), limit of detection (LOD), linearity, accuracy, precision, carry-over, matrix effect, stability, and incurred sample reanalysis (ISR). In addition, recovery from volumetric absorptive microsampling (VAMS) and quantitative dried blood spot (qDBS) devices was evaluated, with specific attention according to the potential influence of the hematocrit effect.

#### 4.7.1. Selectivity and Specificity

Selectivity was evaluated by analyzing 10 replicates of drug-free double blank samples (without stable isotope-labelled internal standard, SIL-IS) and zero calibrator samples (with SIL-IS only) for each matrix. Analyte and internal standard responses were considered acceptable if they did not exceed 20% and 5% of the LLOQ signal, respectively. Specificity was further assessed by examining potential interferences from commonly co-administered drugs, endogenous substances, and known metabolites.

#### 4.7.2. Calibration and Linearity

Calibration curves were prepared over the validated concentration ranges detailed in Table 1, using a linear regression model with a 1/x weighting factor. The relationship between the analyte-to-internal standard peak area ratio and nominal analyte concentrations was assessed for the calibration process. For each matrix, linearity was evaluated using 10 independently prepared calibration curves based on a spiked matrix approach.

All calibration curves met acceptance criteria, with correlation coefficients (*R*) ≥ 0.990 and coefficients of determination (*R*^2^) ≥ 0.995. Additionally, back-calculated concentrations of at least 75% of non-zero calibrators, including the lower limit of quantification (LLOQ) and upper limit of quantification (ULOQ), fell within ±15% of nominal values (±20% for LLOQ), confirming method linearity and accuracy across the validated range [8].

#### 4.7.3. Accuracy and Precision

Intra- and inter-day accuracy and precision were assessed using quality control (QC) samples at four concentration levels: low (LQC), medium (MQC_1_ and MQC_2_), and high (HQC). Each level was analyzed in sextuplicate within a single analytical run (intra-day) and across six independent runs on different days (inter-day). Accuracy was reported as the mean ± standard deviation (SD), while precision was expressed as the coefficient of variation (CV%).

The EMA acceptance criteria are fulfilled when the mean accuracy and CV% are within ±15% of nominal concentrations for all QC levels, and ±20% at the lower limit of quantification (LLOQ) [8].

#### 4.7.4. Carry-Over Effect

Carry-over was evaluated across 10 analytical runs by injecting a high-quality control (HQC) sample, immediately followed by a double blank sample (without analyte or internal standard). Carry-over was considered negligible if the analyte and internal standard (IS) signals in the blank sample did not exceed 20% and 5% of the lower limit of quantification (LLOQ) response, respectively [8,13]. 

#### 4.7.5. Matrix Effect, Process Efficiency, and Extraction Recovery

Matrix effect (ME), process efficiency (PE), and extraction recovery (RE) were evaluated in accordance with the approaches described by Matuszewski et al. and Taylor et al. [15,16]. Three sets of quality control (LQC and HQC) samples (A–C) were prepared in sextuplicate using six independent sources of whole blood and plasma. The sample sets included the following:

- Set A: matrix-free samples spiked post-extraction (neat standard solutions);
- Set B: samples spiked before extraction (to assess recovery);
- Set C: samples spiked after extraction (to assess matrix effect).

The following equations were used for calculation:

- Matrix effect (ME) = (*C*/*A*) × 100%
- Process efficiency (PE) = (*B*/*A*) × 100%
- Extraction recovery (RE) = (*B*/*C*) × 100%

This experimental setup allowed the detailed evaluation of potential ion suppression and enhancement, as well as the overall robustness of the method.

#### 4.7.6. Stability

Stability of everolimus was tested under various conditions, including freeze–thaw cycles, bench-top exposure, autosampler storage, and long-term storage of dried microsamples (VAMS tips and qDBS cards). Analyte concentrations were considered stable if measured values remained within ±15% of their nominal concentrations for each tested condition, in accordance with EMA bioanalytical method validation guidelines [8].

#### 4.7.7. Incurred Sample Reanalysis (ISR)

Incurred sample reanalysis (ISR) was conducted on a minimum of 10% of the total study samples to assess method reproducibility. Reanalyzed concentrations were compared to initial values, and agreement was considered acceptable if at least 67% of the reanalyzed samples fell within ±20% of their original concentration. This evaluation confirmed the consistency and reliability of the method under actual clinical conditions [8]. In accordance with IATDMCT recommendations for method reproducibility, the duplicate sample was collected simultaneously with the first sample [5].

### 4.8. Cross- and Clinical Validation—Correlation Study

Paired sample comparisons were conducted using the Passing–Bablok regression model, Bland–Altman bias analysis, and Pearson correlation to evaluate the data agreement and equivalence between methods. The regression slope was considered acceptable if it remained within ±10% of the theoretical value of 1.0, with 0 included in the confidence interval for the intercept and 1 for the slope. Bland–Altman analysis was used to estimate the mean bias and limits of agreement. Following EMA and IATDMCT recommendations, agreement was accepted when the mean bias remained within ±20% (LoA, analytical limit of agreement) or ±15% (CoA, clinical limit of agreement) for at least 67% of the paired data points [5,8]. For cross-validation and clinical evaluation, comparisons were conducted in accordance with the Measurement Procedure Comparison and Bias Estimation Using Patient Samples guideline [8,19,20,21].

### 4.9. Statistical Analysis

Method validation calculations were performed using Microsoft Excel (v16.91; Microsoft Corp., Redmond, WA, USA). Quantitative results are expressed as mean ± standard deviation (SD), with the coefficient of variation (CV%) reported where applicable. Cross-validation and clinical comparison analyses were conducted using MedCalc software (v23.1.6; MedCalc Software Ltd., Ostend, Belgium), which facilitated Passing–Bablok regression, Bland–Altman bias estimation, and Pearson correlation analyses. A significance level of *p* < 0.05 was applied for all statistical tests, except for correlation analyses, for which *p* < 0.0001 was considered statistically significant.

### 4.10. Hemoglobin Determination in Microsamples and Evaluation of Hematocrit Effect

Although VAMS and qDBS technologies are designed to minimize hematocrit (HCT) bias, assessment of potential hematocrit-related interference remains critical during novel method development and validation. Capillary blood is frequently associated with lower hematocrit values compared to venous whole blood due to a higher plasma fraction. In this study, the influence of hematocrit was evaluated for both microsampling devices using two complementary approaches [3,5]:

- By spiking whole blood samples at three various HCT levels (23%, 37%, and 58%) with two concentrations of everolimus (LQC and HQC), followed by recovery assessment.
- By correlating individual HCT values with the differences in everolimus concentrations between venous whole blood and microsampling devices in clinical samples.

Hematocrit estimation for each patient sample was performed by determining hemoglobin (Hb) concentration using a modified colorimetric assay (Cayman Chemical, Ann Arbor, MI, USA). Hb values were then converted to estimated HCT values by applying a multiplication factor of 3.0. For this purpose, two microsamples were collected per patient—one for EVE quantification (processed with organic extraction) and another for HCT determination (processed in aqueous solution). Further spectrophotometric and assay-specific details are available in the assay kit documentation [30].

## 5. Conclusions

In summary, a robust LC-MS/MS method was successfully developed and thoroughly validated for quantifying everolimus in venous whole blood and capillary blood samples collected using Mitra™ and Capitainer^®^ microsampling devices. The method exhibited excellent analytical performance aligned with regulatory guidelines, with high precision, accuracy, and stability. Its clinical utility was confirmed through successful cross-validation and hematocrit-independent measurements, supporting its suitability for routine therapeutic drug monitoring. Additionally, this approach facilitates decentralized sampling, which is especially beneficial for post-transplant patient care and remote monitoring strategies. To conclude, microsampling offers notable advantages in accessibility, sampling frequency, and overall health outcomes for the SOT population. Less invasive TDM techniques based on microsampling could minimize the need for frequent visits to transplant centres, thereby enhancing access to treatment for remote patients. The capacity for self-sampling at home and the logistics of sample processing to laboratories could enable more frequent remote telemonitoring, allowing for dose adjustments of EVE. Ultimately, this simplified and improved TDM process leads to better graft survival rates, reduced drug toxicity, and lower incidences of graft rejection, thereby improving overall outcomes for SOT patients. 

## Figures and Tables

**Table 1:** Selected strengths and limitations characteristic of Mitra™ and Capitainer^®^ microsampling devices.

Microsampling Device	Strengths	Limitations
Mitra™ (VAMS)	device is enabled to collection of one sample possibility of visual control of sampling correctness	relatively high risk of sample contamination relatively high risk of overloaded the tip
Capitainer^®^ (qDBS)	device is enabled to collection of two samples possibility of visual control of sampling correctness relatively low risk of sample contamination	analytical issues related to the presence of Patent Blue and adhesive components (glue) difficulties related to sampling (in fact a more blood is necessary to fill up the channels and generation of 10 µL spot)

qDBS—quantitative dried blood spot, VAMS—volumetric absorptive microsampling.

**Table 2:** Results of accuracy and precision examination in intra-run (intra-day) and inter-run (inter-day) experiments for five levels of quality controls for whole blood, VAMS, and qDBS samples.

Parameter	LLOQ			LQC			MQC_1_			MQC_2_			HQC		
	WB (1.27 ng/mL)	VAMS (0.50 ng/mL)	qDBS (0.50 ng/mL)	WB (2.36 ng/mL)	VAMS (0.75 ng/mL)	qDBS (0.75 ng/mL)	WB (4.34 ng/mL)	VAMS (3.50 ng/mL)	qDBS (3.50 ng/mL)	WB (8.77 ng/mL)	VAMS (7.50 ng/mL)	qDBS (7.50 ng/mL)	WB (30.0 ng/mL)	VAMS (35.0 ng/mL)	qDBS (35.0 ng/mL)
**Intra-run (intra-day) accuracy and precision [n = 6]**															
C_EVE_ [ng/mL]	1.33 ± 0.11	0.52 ± 0.04	0.52 ± 0.04	2.43 ± 0.22	0.71 ± 0.04	0.72 ± 0.04	4.58 ± 0.30	3.54 ± 0.11	3.45 ± 0.13	8.81 ± 0.48	7.53 ± 0.09	7.57 ± 0.25	30.40 ± 1.34	35.44 ± 2.06	36.40 ± 2.48
Accuracy [%]	106.70	105.55	103.28	101.60	101.64	97.71	101.86	99.66	98.95	99.34	100.02	94.94	99.97	98.98	99.97
Precision [%]	9.67	8.34	7.77	8.89	8.61	7.56	6.65	7.17	7.66	5.42	2.04	6.35	1.40	2.01	1.92
**Between-run (inter-day) accuracy and precision [n = 6]**															
C_EVE_ [ng/mL	1.30 ± 0.10	0.53 ± 0.03	0.51 ± 0.03	2.34 ± 0.14	0.75 ± 0.04	0.73 ± 0.03	4.48 ± 0.23	3.60 ± 0.11	3.65 ± 0.09	8.55 ± 0.29	7.53 ± 0.09	7.57 ± 0.17	29.96 ± 1.39	34.57 ± 1.45	35.40 ± 0.48
Accuracy [%]	98.24	103.44	99.55	98.28	100.64	100.84	98.57	101.43	101.00	101.22	101.02	101.70	100.19	100.75	99.95
Precision [%]	7.95	8.22	6.26	5.90	6.12	5.70	5.09	5.71	4.20	3.38	2.04	2.20	0.73	2.04	1.32

**Table 3:** Results of matrix effect, process efficiency, and absolute recovery parameters for EVE/EVE-D_4_ ratio for whole blood, VAMS, and qDBS samples [n = 6]. Data are expressed as mean with CV%.

Tested Matrix	Matrix Effect (ME) ^1^	Absolute Recovery (AR) ^1^	Process Efficiency (PE) ^1^
WB LQC	0.89 (2.75)	1.03 (2.16)	1.01 (4.87)
WB HQC	1.21 (9.42)	1.11 (11.58)	1.05 (9.79)
VAMS LQC	0.99 (1.96)	0.92 (2.34)	0.93 (3.68)
VAMS HQC	1.06 (11.01)	0.97 (10.99)	0.95 (10.97)
qDBS LQC	0.95 (2.41)	0.91 (2.41)	0.89 (3.99)
qDBS HQC	1.10 (12.06)	0.94 (11.46)	0.91 (11.08)

^1^ ME = B/A, AR = C/B, PE = C/A, where A is the sample without the matrix, B is the sample spiked with the analyte and IS post-extraction, and C is the sample spiked with the analyte and IS before extraction. HQC, higher quality control; LQC, lower quality control; qDBS, quantitative dried spot; VAMS, volumetric absorptive microsampling; WB, whole blood.

**Table 4:** Results of stability examination for VAMS and qDBS samples [n = 3]. Data presented as mean and CV%.

Stability Experiment	24 H [%]	1 Week [%]	1 Month [%]	3 Months [%]	6 Months [%]
AT/VAMS LQC	100.09 (2.10)	97.98 (4.08)	N/T	N/T	N/T
AT/VAMS HQC	99.56 (0.77)	98.99 (1.20)	N/T	N/T	N/T
RT/VAMS LQC	97.99 (1.77)	98.34 (3.33)	96.04 (1.45)	92.45 (2.04)	89.39 (2.69)
RT/VAMS HQC	98.51 (1.01)	98.01 (1.79)	97.23 (0.97)	93.67 (0.99)	90.81 (1.51)
40°/VAMS LQC	99.16 (1.67)	99.23 (1.45)	96.56 (1.37)	93.21 (1.45)	90.14 (1.87)
40°/VAMS HQC	101.34 (0.81)	97.99 (1.22)	98.23 (1.66)	94.67 (2.08)	89.36 (2.98)
60°/VAMS LQC	87.07 (4.49)	83.95 * (6.42)	N/T	N/T	N/T
60°/VAMS HQC	91.21 (3.56)	87.02 * (3.99)	N/T	N/T	N/T
AT/qDBS LQC	102.30 (3.32)	98.43 (3.65)	N/T	N/T	N/T
AT/qDBS HQC	100.11 (3.50)	97.99 (1.58)	N/T	N/T	N/T
RT/qDBS LQC	99.86 (1.68)	97.94 (2.22)	98.03 (3.44)	96.25 (4.46)	93.23 (4.74)
RT/qDBS HQC	101.01 (0.89)	100.87 (1.06)	98.76 (1.18)	97.28 (1.76)	94.56 (2.01)
40°/qDBS LQC	99.98 (2.54)	97.34 (3.89)	95.44 (2.53)	93.22 (2.79)	90.93 (3.59)
40°/qDBS HQC	100.56 (0.99)	98.65 (4.06)	96.71 (2.19)	95.17 (2.42)	91.58 (2.79)
60°/qDBS LQC	90.43 (5.89)	86.42 * (7.44)	N/T	N/T	N/T
60°/qDBS LQC	89.23 (2.60)	85.01 * (2.82)	N/T	N/T	N/T

[%] is the mean sample stability compared to T = 0 (initial conditions), AT—autosampler stability of extract, N/T—not tested, *—stability test ended after 36 h.

**Table 5:** Demographic and clinical data of the patients included in the study.

Variable	Value or Characteristics
Total number of patients	33
Total number of samples (WB/VAMS/qDBS)	66/66/66
Patient’s sex [♂/♀]	18/15
Patient’s age [mean, range]	60.88 (38.67–78.83)
Type of transplantation (Tx) (KTx/LTx/PTx)	(29/2/2)
Hematocrit [%] Hemoglobin [mg/dL]	39.78 (29–51) 13.11 (9.5–16.6)
Creatinine [mg/dL]	1.80 (0.80–3.58)
eGFR (MDRD) [mL/min/m^2^]	38.71 (18–94)
ALT [U/L]	27 (12–82)
AST [U/L]	26 (12–39)
Daily dose of EVE (Certican^®^) [mean, range; mg]	1.82 (0.75–3.00)

ALT, alanine transaminase; AST, aspartate transaminase; EVE, everolimus; KTx, kidney transplantation; LTx, liver transplantation; PTx, pancreas transplantation; qDBS, quantitative dried spot; VAMS, volumetric absorptive microsampling; WB, whole blood.

**Table 6:** Results of cross- and clinical validation of results.

Type of Statistical Test	Evaluated Relationship (Paired Samples) [n = 66]		
	WB-LC-MS/MS Versus VAMS-LC-MS/MS	WB-LC-MS/MS Versus qDBS-LC-MS/MS	VAMS-LC-MS/MS Versus qDBS-LC-MS/MS
Regression formula	EVE_WB_ = 1.017(EVE_VAMS_) − 0.040	EVE_WB_ = 0.938(EVE_qDBS_) + 0.143	EVE_qDBS_ = 1.064(EVE_VAMS_) − 0.193
Intercept (A)	−0.040 (−0.603 to 0.382)	0.1435 (−0.460 to 0.634)	−0.1934 (−0.7829 to 0.2177)
Slope (B)	1.017 (0.923 to 1.127)	0.938 (0.852 to 1.071)	1.0636 (0.9490 to 1.1994)
Mean bias [%]	−0.68 (−4.89 to 3.53)	−1.73 (−6.48 to 3.02)	−1.00 (−5.37 to 3.36)
Residual Standard Deviation (RSD)	0.5022	0.5355	0.5640
% of paired samples fulfilled LoA (mean *bias* < 20%)	83.33%	77.27%	75.76%
% of paired samples fulfilled CoA (mean *bias* < 15%)	69.69%	69.67%	62.12%
*Pearson’s* correlation coefficient (R^2^)	0.935	0.932	0.923
*Spearman* rank correlation coefficient (SRCC) [*p* < 0.0001]	0.887	0.867	0.841

CoA, clinical limit of agreement; LoA, analytical limit of agreement; qDBS, quantitative dried spot; VAMS, volumetric absorptive microsampling; WB, whole blood.

**Table 7:** Nominal EVE concentration values of calibrators defined for whole blood, VAMS, and qDBS samples.

Sample Type	Assigned Concentration [ng/mL]						
	CS_1_ (LLOQ)	CS_2_	CS_3_	CS_4_	CS_5_	CS_6_	CS_7_ (ULOQ)
whole blood (WB)	1.27	2.60	5.36	11.70	24.30	47.80	64.80
Mitra™ (VAMS)	0.50	1.00	2.50	5.00	10.00	25.00	60.00
Capitainer^®^ (qDBS)	0.50	1.00	2.50	5.00	10.00	25.00	60.00

CS—calibrator, LLOQ—lower limit of quantification, qDBS—quantitative dried blood spot, ULOQ—upper limit of quantification, VAMS—volumetric absorptive microsampling.

**Table 8:** Nominal EVE concentration values of quality controls defined for whole blood, VAMS, and qDBS samples.

Sample Type	Assigned Concentration [ng/mL]			
	LQC	MQC_1_	MQC_2_	HQC
whole blood (WB)	2.36	4.34	8.77	30.00
Mitra™ (VAMS)	0.75	3.50	7.50	35.00
Capitainer^®^ (qDBS)	0.75	3.50	7.50	35.00

HQC—higher quality control, LQC—lower quality control, MQC—medium quality control, qDBS—quantitative dried blood spot, VAMS—volumetric absorptive microsampling.

**Table 9:** Multiple reaction monitoring (MRM) parameters for everolimus and corresponding internal standard.

Analyte	Monitored Adduct	Q1 [m/z]	Q3 [m/z]	DP [eV]	CE [eV]	EP [eV]	CXP [eV]
EVE-1 (quantitative pair)	[M + NH_4_]^+^	975.60	908.40	50	25	15	5
EVE-2 (qualitative pair)		975.60	926.70	70	25	15	5
D_4_-EVE-1 (quantitative pair)		979.60	912.50	50	20	15	5
D_4_-EVE-2 (qualitative pair)		979.60	930.60	50	25	15	5

CE—collision energy, CXP—collision cell exit potential, DP—declustering potential, EP—exit potential, EVE—everolimus, D_4_-EVE—deuterated everolimus, Q1—precursor ion, Q3—product ion.

## Data Availability

The data presented in this study are available on request from the corresponding author.

## Appendix A

The influence of hematocrit on analyte recovery was assessed using the VAMS and qDBS samples with 0.23 (23%), 0.37 (37%), and 0.58 (58%) hematocrit values (L/L), spiked with LQC and HQC EVE levels. The calculated results were normalized to IS and expressed as recovery range with CV% and presented in the Table A1. Acceptance criteria have been fulfilled—mean recovery in 85–115% range, CV% < 15%.

**Table A1:** Results of potential hematocrit evaluation in qDBS and VAMS samples [n = 3]. The recovery expressed as mean ± SD with CV%.

Type of Tested Sample	Hematocrit Value		
	0.23	0.37	0.58
qDBS (LQC)	89.63 ± 2.97 (3.32)	91.63 ± 1.67 (1.82)	87.96 ± 2.51 (2.85)
qDBS (HQC)	98.57 ± 0.52 (0.62)	98.41 ± 0.94 (0.94)	92.78 ± 1.74 (1.61)
VAMS (LQC)	90.10 ± 2.06 (2.35)	87.41 ± 2.06 (2.35)	88.23 ± 2.51 (1.99)
VAMS (HQC)	97.41 ± 0.51 (0.56)	96.10 ± 0.41 (0.67)	96.33 ± 0.91 (0.85)

qDBS–quantitative dried blood spot; VAMS—volumetric absorptive microsampling; LQC—lower quality control (0.75 ng/mL); HQC—higher quality control (35 ng/mL).
